# Laccase Production from a Temperature and pH Tolerant Fungal Strain of *Trametes hirsuta* (MTCC 11397)

**DOI:** 10.1155/2013/869062

**Published:** 2013-04-24

**Authors:** Kusum Dhakar, Anita Pandey

**Affiliations:** G. B. Pant Institute of Himalayan Environment and Development, Kosi-Katarmal, Almora, Uttarakhand 263 643, India

## Abstract

Laccase production by a temperature and pH tolerant fungal strain (GBPI-CDF-03) isolated from a glacial site in Indian Himalayan Region (IHR) has been investigated. The fungus developed white cottony mass on potato dextrose agar and revealed thread-like mycelium under microscope. ITS region analysis of fungus showed its 100% similarity with *Trametes hirsuta*. The fungus tolerated temperature from 4 to 48°C ± 2 (25°C opt.) and pH 3–13 (5–7 opt.). Molecular weight of laccase was determined approximately 45 kDa by native PAGE. Amplification of laccase gene fragment (corresponding to the copper-binding conserved domain) contained 200 bp. The optimum pH for laccase production, at optimum growth temperature, was determined between 5.5 and 7.5. In optimization experiments, fructose and ammonium sulfate were found to be the best carbon and nitrogen sources, respectively, for enhancing the laccase production. Production of laccase was favored by high carbon/nitrogen ratio. Addition of CuSO_4_ (up to 1.0 mM) induced laccase production up to 2-fold, in case of 0.4 mM concentration. Addition of organic solvents also induced the production of laccase; acetone showed the highest (2-fold) induction. The study has implications in bioprospecting of ecologically resilient microbial strains.

## 1. Introduction

Laccases (phenol oxidases; E.C. 1.10.3.2.), also known as multicopper blue oxidases, belong to the oxidoreductase group of enzymes. Biochemically, they are glycoproteins carrying molecular mass between 50 kDa and 130 kDa [1]. Fungi, belonging to ascomycetes, deuteromycetes, and basidiomycetes, are known to produce laccases of ecological as well as biotechnological importance, such as biodegradation and bioremediation [1–4]. In addition, laccases are also responsible for various physiological functions in fungi [5]. Due to their broad specificity toward substrate, they can oxidize a range of chemical compounds leading to various industrial applications [6]. 

 Enhancement of laccase production, by modifying the nutritional and physiological conditions during cultivation of promising fungi, is a prerequisite for their optimum utilization at industrial scale. Besides nutritional supplements, inducers like organic solvents and metal ions also play important role in production of laccases [3, 7, 8]. Isolation of new microbial strains of biotechnological applications from various ecological habitats is a prerequisite for industrial growth. The fungi capable of producing laccase at wider temperature and pH range are likely to play important role in biodegradation under low temperature environments. In the present study, a temperature and pH tolerant fungus isolated from a glacial site in Indian Himalayan Region (IHR) has been investigated for production of laccase at different physicochemical and nutritional conditions.

## 2. Materials and Methods

### 2.1. Fungal Strain (GBPI-CDF-03)

The fungus was originally isolated from the soil collected from alpine zone of Pindari glacier region (33°5′-30°10′N to 70°48′-79°52′E), covering altitudes between 3000 and 4500 m amsl (above mean sea level) of IHR. The mean monthly temperature, at the site, has been reported between 5.5°C (January) and 20.1°C (August); the soil pH ranged from 4.5 to 5.1. The description of site has been reported earlier [9–11]. The soil was serially diluted and appropriate dilution was plated using potato dextrose agar (PDA). The pure fungus obtained was maintained on PDA slants following subculturing (2 months interval) at 4°C, in the Microbial Culture Collection established in the Microbiology Laboratory of the Institute. Fresh culture was raised for the experimentation.

### 2.2. Identification and Characterization of the Fungus

Colony morphology of the fungus was recorded on 5 days old culture, grown on PDA at 25°C. Four other media, namely, Mycological agar, Czapek dox agar, Sabouraud dextrose maltose agar and Vegetable juice 8 agar (all from Hi Media), were also used for recording the observations on the growth of fungus. Microscopic observations were recorded following lactophenol cotton blue staining (Nikon-Eclipse 50i, Japan). Molecular identification was based on ITS (ITS1-5.8S-ITS2) region analysis using ITS1 and ITS4 primers [12]. The fungal isolate and the gene sequence have been accessioned in Microbial Type Culture Collection and Gene Bank, Institute of Microbial Technology, Chandigarh, India, and NCBI, respectively. The phylogenetic tree was made by using NJ method with the bootstrap value 1000. MEGA 4.0 was used for the phylogenetic analyses (courtesy: A. Sharma, MCC, NCCS, Pune, India). 

 Temperature tolerance was determined on PDA by incubating the fungal strain between 4 and 50°C (4, 9, 14, 25, 35, 45, and 50°C), up to two weeks. The pH tolerance was determined between 1 to 14 pH (with 0.5 pH interval), by incubating the fungus at 25°C for one week on PD agar/broth. 

### 2.3. Medium and Culture Condition for Laccase Production

Laccase production was carried out using modified Kirk and Farrell [13] medium containing (g/L) 2.0 g malt extract, 2.0 g glucose, 2.0 g NH_4_NO_3_, 0.26 g Na_2_HPO_4_, 0.26 g KH_2_PO_4_, 0.5 g MgSO_4_ (7H_2_O), 0.01 g CuSO_4_ (5H_2_O), 0.006 g CaCl_2_ (2H_2_O), 0.005 g FeSO_4_ (7H_2_O), 0.0005 g ZnSO_4_ (7H_2_O), 0.00002 g Na_2_MoO_4_, 0.00009 g MnSO_4_·H_2_O, and 0.00007 g H_3_BO_3_. Qualitative plate based estimation was done on the aforesaid medium supplemented with 0.30 g ABTS (2, 2′-azino-bis 3-ethylbenzothiazoline-6-sulphonic acid). The plate assays were carried out at 6 different temperatures (4, 9, 15, 25, 35, and 45°C). Ligninolytic efficiency was determined as ABTS zone to fungal colony diameter ratio ∗ 100. DMP, syringaldazine, and guaiacol (separately) were also used in plate assays for determination of the oxidizing ability of laccase. In quantitative estimations, 50 mL medium was prepared in 250 mL Erlenmeyer flasks, without supplementing ABTS. The pH of the medium was set at 5.5 ± 0.2, before autoclaving. Autoclaved medium was inoculated with 5 mm disc (per flask) of 6-day old fungus culture. The experiments were conducted under static conditions.

### 2.4. Determination of Molecular Weight of Laccase

The crude enzyme (filtrate) was precipitated with ammonium sulfate up to 70% saturation and centrifuged at 15000 ∗ g RCF for 10 min at 4°C. Pellets were resuspended in citrate-phosphate (pH = 2.6) buffer. PAGE without SDS was carried out following standard method described by Laemmli [14], using 15% separating and 4% stacking gel. Polyacrylamide gel was stained with 1.0% of ABTS solution prepared in citrate-phosphate buffer (pH 2.6), following 1 h incubation at room temperature. Pre-stained protein marker (Puregene) was used for determination of molecular weight of the enzyme.

### 2.5. Molecular Studies on Partial Sequence of Laccase Gene

Laccase gene fragment (corresponding to conserved copper binding domain) was amplified by PCR (BIO-RAD, USA). DNA isolation was done by the method given by Voigt et al. [15]. PCR amplification was carried out using basidiomycetes specific laccase primers Cu1F and Cu2R [16]. 25 *μ*L PCR reaction contained 2X reaction buffer 12.5 *μ*L, sterile water 6.2 *μ*L, dNTP (10 mM of each dNTP) 2.5 *μ*L, MgCl_2_ (25 mM) 1.5 *μ*L, forward and reverse primer 1.0 *μ*L (10 pmole/*μ*L each), and Taq DNA polymerase (3 U/*μ*L) 0.3 *μ*L (dNTP, MgCl_2_, and Taq DNA polymerase) from Fermentas Life Science. The PCR conditions were initial denaturation: 94°C for 3 min, denaturation: 94°C for 30 s, annealing: 48°C for 45 s, extension: 72°C for 3 min, final extension: 72°C for 10 min, and total cycles: 35. Molecular weight of amplified product was analyzed on agarose gel (0.8%). 

### 2.6. Effect of Physicochemical and Nutritional Parameters and Other Supplements on Laccase Production

Production of laccase was determined at 4 temperatures (15, 25, 35, and 45°C) at every 3rd day, up to 15 days of incubation. Laccase production with respect to pH was determined by inoculating the broth, set at different pH, ranging from 3.5 to 11.5 (with an interval of 2 units). 

Six carbon, glucose (control), fructose, maltose, sucrose, starch and cellulose, 5 nitrogen sources (ammonium nitrate (control), ammonium sulfate, ammonium ferrous sulfate, potassium nitrate, and urea), and one set without inorganic nitrogen at 0.2% level were used for enhancing the production of laccase. All the carbon and nitrogen sources were from Hi Media. Effect of the best carbon and nitrogen sources was further determined by increasing the concentration level up to 1.0%.

In other supplements category, 4 organic solvents namely, methanol, ethanol, isopropanol, and acetone (0.2%/supplemented at day 4 of incubation), were tested as inducers. Effect of CuSO_4_ concentration (0.2 to 1.0 mM/supplemented at day 4 of incubation) was also determined.

These experiments (effect of pH, carbon sources, nitrogen sources, CuSO_4_, and solvents) were carried out at 25°C, following 12 days of incubation under static conditions. 

### 2.7. Enzyme Assay

Laccase activity was determined in terms of oxidation of ABTS using spectrophotometer (Amersham Bioscience Ultrospec 2100 pro) [17]. The fungal culture was filtered with Whatman No. 1 filter paper and used as crude enzyme. Mycelium, collected and dried at 65°C for 72 h, was used for determination of biomass. Reaction mixture was prepared with citrate-phosphate buffer (pH = 2.6), 2.0 mM ABTS, and the crude enzyme. Absorbance was recorded at 420 nm, following 2 min of incubation at room temperature. Laccase activity was calculated using extinction coefficient = 36000 M^−1^ cm^−1^ [18]. 1 unit of enzyme activity is defined as 1 *μ*M of ABTS oxidized per min. Protein was also determined following the method of Lowry et al. [19].

## 3. Results 

**Figure 1:**
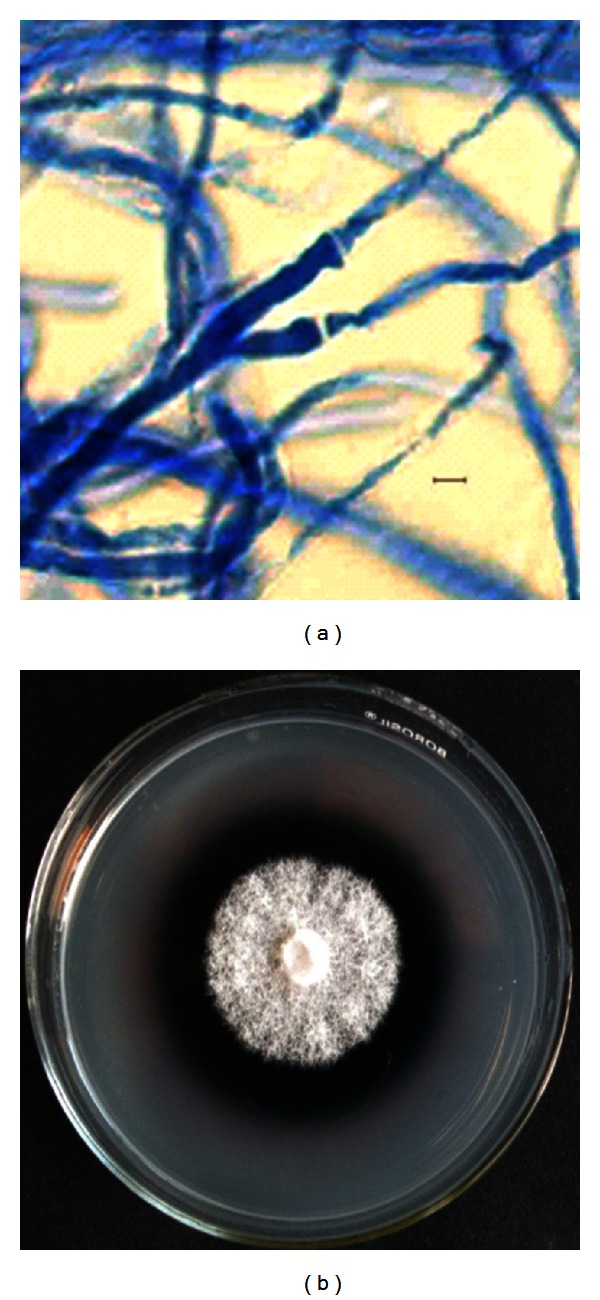
(a) Microscopic features of the fungus (Bar = 2 *μ*m); (b) laccase production on ABTS plate.

**Figure 2:**
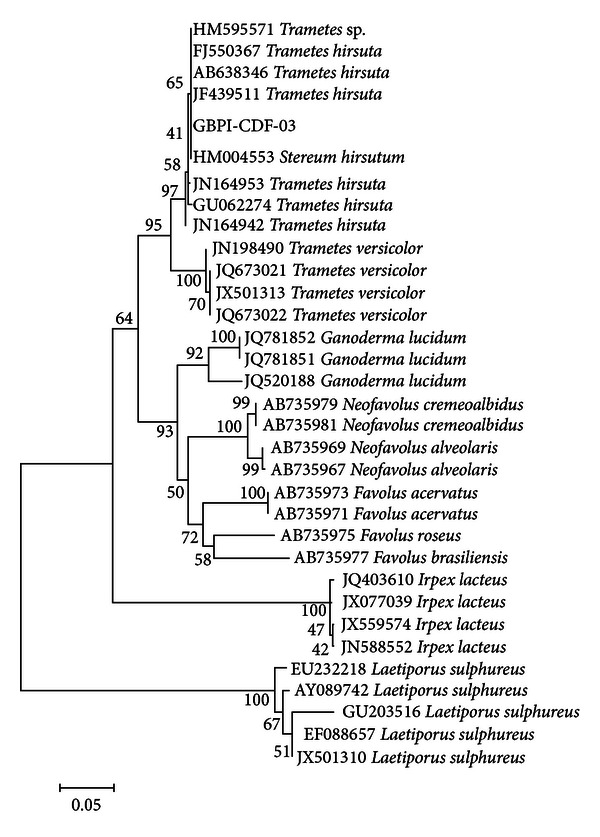
Phylogenetic relationship of the fungus.

The fungus (GBPI-CDF-03) developed white cottony mass on all the 5 media used, with growth being more compact on Vegetable juice 8 agar. Microscopy revealed presence of thread-like septate mycelium without any reproductive structures (Figure 1(a)). The fungus tolerated wide range of temperature (4–48 ± 2°C, optimum 25 ± 2°C) and pH (3.0–13.0 pH, optimum between 5 and 7). ITS region based molecular identification revealed its maximum similarity with *Trametes hirsuta*. Growth requirements along with the phenotypic and genotypic characters and the phylogenetic relationship of the fungus are presented in Table 1 and Figure 2. The accessions given to fungus and the nucleotide sequence are MTCC 11397 and JX910367, respectively. 

**Figure 3:**
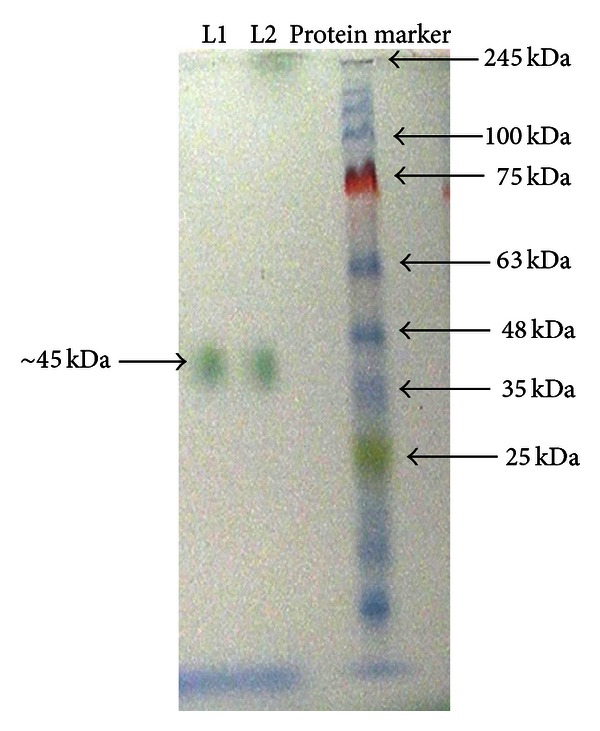
Zymogram of laccase (L1 and L2) stained with 1% ABTS following native PAGE, M-prestained protein marker.

**Figure 4:**
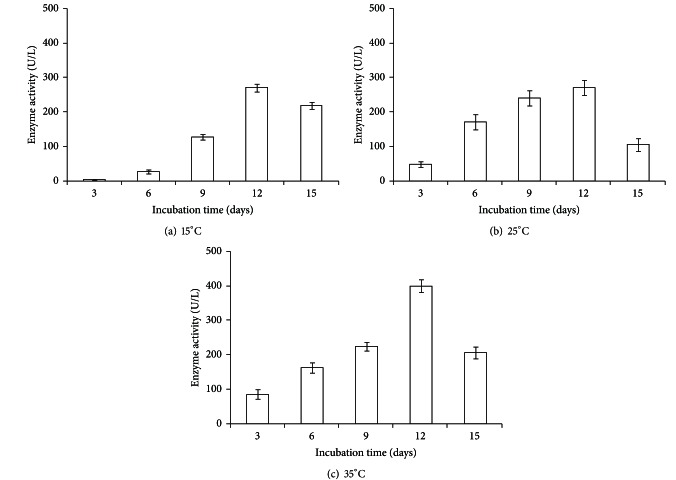
Effect of different temperatures with respective time on laccase production: (a) 15°C, (b) 25°C, (c) 35°C.

**Figure 5:**
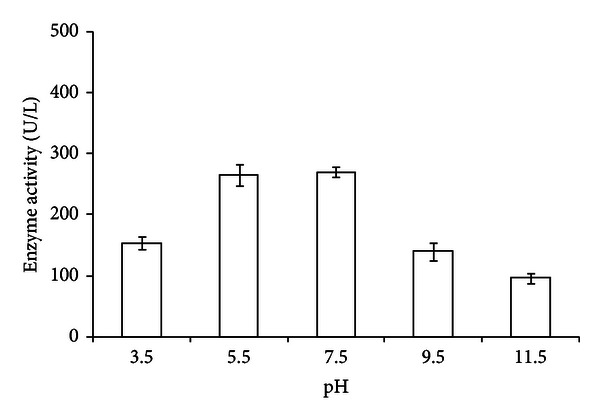
Effect of pH on laccase production.

In plate assays, conducted at optimum growth conditions, the fungus oxidized ABTS and developed green colour around the colony indicating the production of laccase (Figure 1(b)). The fungus also produced laccase through oxidation of other three substrates, namely, guaiacol, syringaldazine, and DMP. ABTS, however, was found to exhibit highest sensitivity towards laccase activity. At suboptimal temperatures (4, 9, 15, 35, and 45°C), maximum ligninolytic efficiency (ABTS zone to colony dia) was observed at 4°C. The efficiency for production of ligninolytic enzymes was found to be inversely proportional to the temperature (Table 2). Molecular weight of laccase was determined to be approximately 45 kDa (Figure 3), while the PCR amplified sequence of laccase gene fragment (corresponding to copper binding domain) was obtained approximately of 200 bp size. In quantitative estimations conducted at 15, 25, 35, and 45°C, production of laccase, fungal biomass, and total protein content varied with culture conditions. Maximum laccase production (394.5 U/L) was recorded at 35°C on day 12 of incubation. In this case, the highest biomass (22.8 mg/50 mL) and maximum protein (51.1 *μ*g/mL) were recorded on day 12 and 15, respectively. Laccase production was recorded minimum (269.7 U/L) at 25°C with maximum fungal biomass (25.6 mg/50 mL) and protein concentration (41.9 *μ*g/mL), at day 12 of incubation. Slightly increased production of laccase (272 U/L) was recorded at 15°C with fungal biomass (14.4 mg/50 mL) and protein content (66.2 *μ*g/mL), at day 12 of incubation (Table 3, Figures 4(a), 4(b), and 4(c)). The activity at 45°C was estimated to be 282.1 U/L with very little biomass (8.0 mg/50 mL) and protein content (16.8 *μ*g/mL), at day 12 of incubation (not presented in the Figures). While the production of laccase was recorded between pH 3.5 and 11.5, the maximum production (270 U/L) along with biomass and the protein content was favoured at pH between 5.5 and 7.5 (Table 4, Figure 5). 

**Figure 6:**
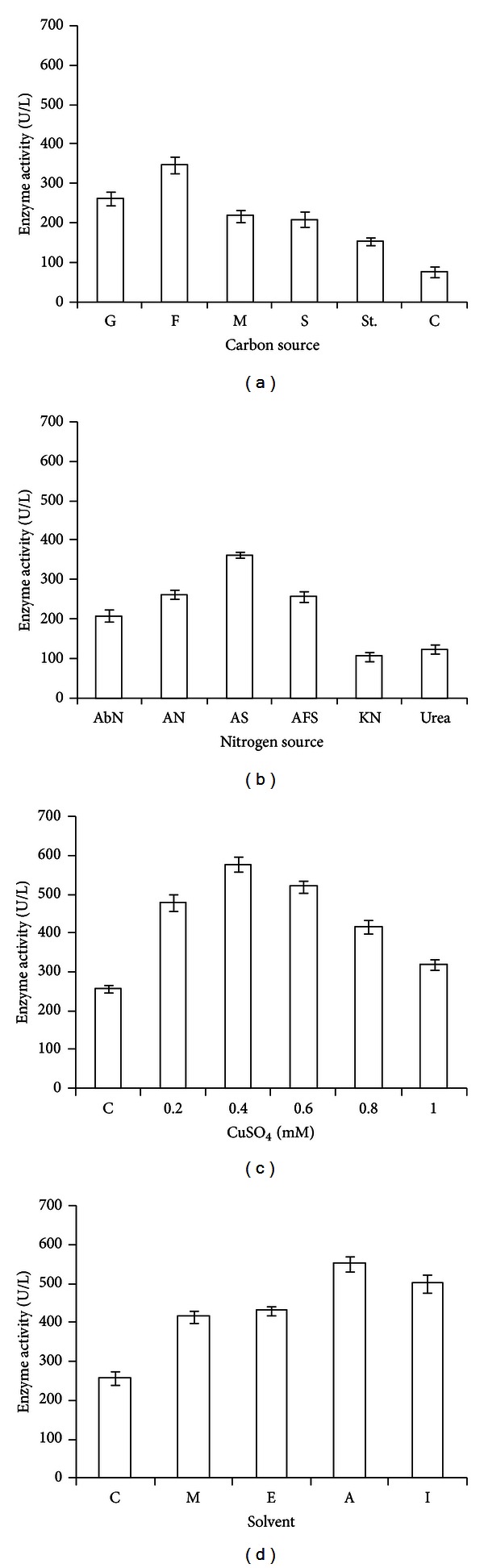
Effect of nutritional parameters on laccase production: (a) carbon sources: G (glucose), F (fructose), M (maltose), S (sucrose), St. (starch), C (cellulose); (b) nitrogen sources: AbN (absence of inorganic nitrogen), AN (ammonium nitrate), AS (ammonium sulfate), AFS (ammonium ferrous sulfate), KN (potassium nitrate); (c) CuSO_4_ concentration (0.2 to 1.0 mM) (d) organic solvents: C (control), M (methanol), E (ethanol), A (acetone), I (iso-propanol).

Among 6 carbon sources (0.2%), fructose was found to increase the production of laccase along with higher protein content and reduced fungal biomass, in comparison to control (Table 5, Figure 6(a)). The laccase production increased with the increase of fructose concentration up to 1.0%, maximum being at 0.4% concentration of fructose (2 : 1 carbon/nitrogen ratio) (Table 6). However, the increase in biomass and protein continued up to 1.0% fructose concentration. Other carbon sources (maltose, sucrose, starch, and cellulose) were found to be inhibitory for laccase production as well as the production of fungal biomass. Cellulose was found to be the most inhibitory carbon source, followed by starch, sucrose, and maltose. Among five nitrogen sources, ammonium sulfate was found to be the best at 0.2% for production of laccase and the protein content (Table 5, Figure 6(b)). Further increment in ammonium sulfate concentration was found to be inhibitory for laccase production (Table 6). Effect of ammonium ferrous sulfate was found to be at par to the control (ammonium nitrate). Presence of other nitrogen sources (urea and potassium nitrate) and also the case without any nitrogen source were found to reduce the production of laccase.

Addition of CuSO_4_ increased laccase production along with production of protein content up to 2-fold in comparison to control, maximum being in case of 0.4 mM concentration. Further increment in CuSO_4_ (up to 1.0 mM) resulted in decline of laccase production, although, the decreased values were higher in comparison to control (Figure 6(c), Table 7). Other inducers, the low molecular weight organic solvents at 0.2% concentration, were also found to enhance the production of laccase, in comparison to control (Figure 6(d), Table 7). Among these, acetone favoured the production of laccase (up to 2 fold induction), with higher protein content. The induction effect, in this case, was recorded up to 0.6% (Table 8). Addition of acetone did not favor the production of fungal biomass.

## 4. Discussion

The growth characters, such as tolerance to wider range of temperature and pH, are indicative of the ecological resilience possessed by the fungus. Isolation of new fungal strains from low temperature environments possessing similar growth characteristics along a range of biotechnological applications has been reported in recent years [20–25]. Temperature and pH are two of the most important factors in production of enzymes. Role of suboptimal temperatures, both towards higher as well as lower (15 and 35°C, resp.), in enhancing the production of laccase was clearly demonstrated in the present investigation. In both the instances, it coincided with decrease in production of fungal biomass. Varying results, based on the organisms as well as the growth conditions, have been reported by different workers on production of ligninolytic enzymes. Šnajdr and Baldrian [26] reported production of laccase by *T. versicolor* between 25 and 30°C, while in a recent study, laccase production has been reported to decrease beyond 28°C [27]. Generally, a bell-shaped curve has been reported for laccase activity with respect to pH [3]. Janusz et al. [28] have reported pH 7.5, optimum for production of laccase by *Rhizoctonia praticola*, as an exceptional case. In a recent study on *Trametes trogii*, pH 3 was found to be optimum for laccase production [29]. 

Optimization for enzyme production can be regulated by modifying the nutritional sources, carbon and nitrogen, in particular. Generally, glucose is considered to be the best carbon source for production of enzymes [7]. Production of laccase, favoured by replacing glucose with fructose in the present study, can be attributed to the specific preference for the carbon source by the fungus under study. Similar observation in case of* Agaricus* sp. has been reported recently by Manimozhi and Kaviyarasan [30]. Among nitrogen sources, ammonium sulfate at 0.2% concentration gave maximum production of laccase, in the present investigation. Reduction in laccase production, on increasing the ammonium sulfate concentration beyond 1 : 1 carbon/nitrogen ratio, can be attributed to the fact that nitrogen depletion is likely to favour the production of laccase [31]. In general, high carbon : nitrogen ratio favors the production of laccase, as observed in the present investigation. Contrary to these observations, reverse conditions have also been reported [32]. 

CuSO_4_ and some organic solvents are known as inducers for enhancing the laccase activity. In the present investigation, CuSO_4_ induced laccase production, almost up to 2 fold, over control. Inducing effect of CuSO_4_ has also been reported in earlier studies in *T. hirsuta*. CuSO_4_ is a constituent of the catalytic center of the laccase; hence it is important for the synthesis of the respective enzyme [33]. Importance of copper in regulation of laccase production at transcriptional levels has also been reported [7]. Enhancement in laccase production, in the present investigation, due to addition of organic solvents, such as, methanol, ethanol, acetone, and isopropanol, is also an important finding. The increase in laccase production by ethanol has been observed in earlier studies [34]. The purpose of using simple organic solvents for induction is to increase the ecological and economical value of the process. 

## 5. Conclusion

It can be concluded that increased production of laccase at suboptimal conditions, as recorded in the present study, is likely to be advantageous from ecological as well as biotechnological prospects. Temperature tolerant organisms, psychrotolerant ones in particular, can be presumed for their contributions in biodegradation in low temperature environments. High yield, consistent production, and sustainability of secondary metabolites under stress conditions will be important parameters in such investigations. The fungus (GBPI-CDF-03) produced laccase under wide range of temperature covering psychrophilic to mesophilic range, also touching the thermophilic range. The present investigation emphasizes the importance of isolation and identification of new and efficient microbial strains from the extreme environments of IHR for production of industrially desired biomolecules [35]. The findings of the present study, based on the identification of a laccase producing fungal strain, followed by the optimization for nutritional and physiological conditions, are likely to be useful in further upgrade of the process. 

## Figures and Tables

**Table 1:** Morphological and molecular characteristics of the fungus (GBPI-CDF-03).

Character	Description
Colony morphology (on PD agar)	White cottony mass with no exudation or pigmentation

Microscopic features	Thread like mycelium with septa showing the characteristic feature of upper fungi

Growth characters	Temperature requirement between 4 and 48°C (optimum 25°C), pH requirement between 3 and 13 (optimum 5–7)

Phylogenetic relationship (ITS region analysis) Culture accession no. Nucleotide sequence accession no.	Maximum similarity (100%) with *Trametes hirsuta *(JF439511) MTCC 11397 JX910367

**Table 2:** Ligninolytic efficiency of the fungus (GBPI-CDF-03) at different temperatures.

Temperature (°C)	Incubation time (days)	ABTS zone dia (mm) (ZD)	Fungal colony dia (mm) (CD)	Ligninolytic efficiency = ZD/CD ∗ 100
4	21	22	5	440
9	21	19	7	271
15	14	15	10	150
25	7	10	50	20
35	7	12	46	26
45	7	8	15	53

Dia: diameter.

**Table 3:** Production of fungal biomass and protein content at different temperatures.

Incubation time (days)	Temperature (°C)					
	15		25		35	
	Biomass (mg/50 mL)	Protein (*μ*g/mL)	Biomass (mg/50 mL)	Protein (*μ*g/mL)	Biomass (mg/50 mL)	Protein (*μ*g/mL)
3	4.1 ± 0.5	36.4 ± 3.1	8.6 ± 1.6	34.4 ± 6.6	5.3 ± 1.1	34.6 ± 5.0
6	7.6 ± 0.8	44.6 ± 10.7	13.0 ± 3.2	36.9 ± 8.2	13.6 ± 2.0	39.4 ± 4.0
9	11.5 ± 2.0	54.6 ± 4.4	17.9 ± 6.2	32.9 ± 4.1	18.7 ± 4.4	42.9 ± 8.0
12	14.4 ± 2.2	66.2 ± 5.7	25.6 ± 4.2	41.9 ± 6.7	22.8 ± 2.8	43.6 ± 5.5
15	17.9 ± 3.4	68.3 ± 5.8	19.8 ± 4.0	33.8 ± 5.5	19.3 ± 2.4	51.1 ± 8.8

Values are mean ± SD (*n* = 3).

**Table 4:** Production of fungal biomass and protein content at different pH.

pH	Biomass (mg/50 mL)	Protein (*μ*g/mL)
3.5	15.3 ± 3.5	19.6 ± 3.9
5.5	24.3 ± 4.5	28.0 ± 2.1
7.5	21.3 ± 3.0	35.1 ± 4.4
9.5	14.0 ± 2.0	30.9 ± 2.8
11.5	8.4 ± 2.6	11.12 ± 2.9

Values are mean ± SD (*n* = 3).

**Table 5:** Production of fungal biomass and protein content in presence of different carbon and nitrogen sources.

Carbon source (0.2%)	Biomass (mg/50 mL)	Protein (*μ*g/mL)	Nitrogen source (0.2%)	Biomass (mg/50 mL)	Protein (*μ*g/mL)
Glucose	24.9 ± 4.0	27.0 ± 5.5	Absence of nitrogen	21.0 ± 2.2	28.6 ± 4.9
Fructose	19.3 ± 3.0	42.3 ± 4.0	Ammonium nitrate	28.0 ± 4.5	34.6 ± 4.5
Maltose	15.6 ± 4.0	33.6 ± 5.5	Ammonium sulfate	23.0 ± 3.6	40.0 ± 5.0
Sucrose	11.3 ± 2.6	39.0 ± 6.5	Ammonium ferrous sulfate	18.5 ± 2.5	38.3 ± 4.5
Starch	10.5 ± 1.8	31.6 ± 5.1	Potassium nitrate	25.1 ± 4.5	19.6 ± 3.2
Cellulose	9.5 ± 1.0	26.1 ± 7.6	Urea	16.4 ± 4.1	41.6 ± 4.0

Values are mean ± SD (*n* = 3).

**Table 6:** Laccase activity, fungal biomass, and protein content with different concentrations of fructose and ammonium sulfate.

Fructose (%)	Laccase (U/L)	Biomass (mg/50 mL)	Protein (*µ*g/mL)	Ammonium sulfate (%)	Laccase (U/L)	Biomass (mg/50 mL)	Protein (*µ*g/mL)
Control	248.0 ± 6.5	22.4 ± 6.8	31.6 ± 4.0	Control	260.0 ± 8.9	26.1 ± 4.4	22.3 ± 4.9
0.2 (1 : 1)	335.0 ± 14.3	28.7 ± 1.5	45.2 ± 6.6	0.2 (1 : 1)	344.0 ± 14.1	29.6 ± 4.5	38.3 ± 8.5
0.4 (2 : 1)	435.0 ± 35.5	31.8 ± 3.6	65.3 ± 8.7	0.4 (1 : 2)	194.0 ± 23.6	27.2 ± 4.4	33.6 ± 7.7
0.6 (3 : 1)	374.0 ± 31.2	34.3 ± 4.0	69.6 ± 4.5	0.6 (1 : 3)	154.0 ± 4.7	25.5 ± 2.2	32.3 ± 3.7
0.8 (4 : 1)	336.0 ± 11.8	36.5 ± 3.4	84.3 ± 10.0	0.8 (1 : 4)	133.0 ± 7.1	24.6 ± 5.0	30.6 ± 3.5
1.0 (5 : 1)	310.0 ± 9.4	37.3 ± 4.5	108.6 ± 13.3	1.0 (1 : 5)	130.0 ± 3.3	25.8 ± 4.9	26.0 ± 4.3

Values given in parentheses depict the carbon/nitrogen ratio; values are mean ± SD (*n* = 3).

**Table 7:** Production of fungal biomass and protein content in presence of different concentrations of CuSO_4_ and different solvents (0.2%).

CuSO_4_ (mM)	Biomass (mg/50 mL)	Protein (*μ*g/mL)	Solvent (0.2%)	Biomass (mg/50 mL)	Protein (*μ*g/mL)
0.2	16.8 ± 2.5	35.6 ± 6.6	Control	25.3 ± 3.0	23.9 ± 3.4
0.4	13.1 ± 3.3	44.0 ± 6.5	Methanol	16.1 ± 3.6	38.1 ± 4.0
0.6	11.7 ± 0.9	46.6 ± 7.0	Ethanol	16.6 ± 2.0	41.8 ± 5.7
0.8	8.3 ± 3.0	54.0 ± 5.5	Acetone	18.4 ± 2.2	45.2 ± 6.1
1.0	7.1 ± 2.5	55.1 ± 7.2	Isopropanol	13.0 ± 2.5	38.3 ± 5.6

Control value for biomass = 24.0 ± 3.8, and protein = 31.2 ± 2.5; values are mean ± SD (n = 3).

**Table 8:** Laccase activity, fungal biomass, and protein content with different concentrations of acetone.

Acetone (%)	Laccase (U/L)	Biomass (mg/50 mL)	Protein (*μ*g/mL)
Control	263 ± 9.4	21.8 ± 3.8	25.1 ± 3.6
0.2	542 ± 1.6	17.7 ± 1.5	45.4 ± 5.9
0.4	397 ± 1.4	17.0 ± 3.0	41.1 ± 6.0
0.6	349 ± 1.0	16.3 ± 4.0	41.0 ± 6.0
0.8	227 ± 1.1	13.3 ± 3.5	35.7 ± 4.5
1.0	201 ± 1.7	11.0 ± 3.0	31.5 ± 4.0

Values are mean ± SD (*n* = 3).
